# Relationships, resources, and political empowerment: community violence intervention strategies that contest the logics of policing and incarceration

**DOI:** 10.3389/fpubh.2023.1143516

**Published:** 2023-04-17

**Authors:** Mia Karisa Dawson, Asia Ivey, Shani Buggs

**Affiliations:** ^1^Geography, University of California, Davis, Davis, CA, United States; ^2^Sociology, University of California, Davis, Davis, CA, United States; ^3^Health, University of California, Davis, Davis, CA, United States

**Keywords:** community violence prevention and intervention, policing, incarceration, safety nets, structural determinants of health, health equity, resource distribution, language and narrative

## Abstract

Community violence—defined as unsanctioned violence between unrelated individuals in public places—has devastating physical, psychological, and emotional consequences on individuals, families, and communities. Immense investments in policing and incarceration in the United States have neither prevented community violence nor systemically served those who have been impacted by it, instead often inflicting further harm. However, the logics that uphold policing and incarceration as suitable or preventative responses to community violence are deeply ingrained in societal discourse, limiting our ability to respond differently. In this perspective, we draw from interviews with leading voices in the field of outreach-based community violence intervention and prevention to consider alternative ways to address community violence. We begin by demonstrating that policing and incarceration are distinguished by practices of retribution, isolation, and counterinsurgency that are counterproductive to the prevention of community violence. Then, we identify alternative practices of outreach-based community violence intervention and prevention that include (1) fostering safety nets through relationships among individuals, families, and neighborhoods, (2) fighting poverty and increasing access to resources, and (3) building political capacity among organizations to transform the broader systems in which they are embedded. They also include accountability practices that are preventative and responsive to the needs of those who are harmed. We conclude that elevating the language, narratives, and values of outreach-based community violence intervention and prevention can transform our responses to violence, interrupt cycles of harm, and foster safer communities.

## Introduction

The United States has consistently ranked last among countries in the Global North in a wide variety of indicators of health and safety (1, 2). Structural racism, income inequality, segregation, and incarceration have each been shown to contribute to universally negative outcomes in areas including environmental health, food security, and life expectancy (3–6). Furthermore, despite massive investments in policing and incarceration, homicide rates are drastically higher in the U.S. than in other Global North countries, driven especially by gun violence (7, 8). In understanding such negative health and safety outcomes and working to remedy them, an expanding body of literature in public health has turned to a focus on structural determinants of health, considering how health outcomes are embedded in “the political and economic organization of our social world” (9). Violence has increasingly been recognized as a public health crisis that is shaped by structural determinants (10).

In major cities across the U.S., gun violence remains significantly higher than it was prior to the COVID-19 pandemic (11). Violence, and gun violence in particular, is a major threat to public health and safety and lingers at the forefront of political discourse. As is the case when tackling most social and public health issues, there is ample debate about the best forms of intervention and prevention. Policymakers argue over who and what is most responsible for the increased rates of violence, and more money and resources have been allocated to curb it. These conversations and allocations of resources are shaped by the narratives that guide our society's views on what drives violence and how it should be addressed.

Responding to violence is widely considered to be the exclusive domain of the criminal legal system through practices of policing, sentencing, and incarceration. Community leaders and advocates around the country have expressed growing concerns that the return of the tough-on-crime rhetoric that expanded the reach of these systems in the 1980s and 1990s, which led the U.S. to become the global “epicenter of mass incarceration” (12), will undermine efforts to reduce the nation's propensity for harsh, harmful responses that meet violence with violence. The backlash against calls to cut police funding following the 2020 George Floyd uprising has contributed to this concern (13).

Community violence intervention and prevention (CVI) is a multi-faceted approach to curbing community violence, defined as unsanctioned violence between unrelated individuals in public places (14). The field of CVI takes an approach to addressing violence that shares more in common with the methods of public health than with those of the criminal legal system. These commonalities include a focus on systemic issues, identification of risk factors, upstream intervention and prevention, and an iterative assessment of effectivity and course correction (15). These strategies resonate with Hemenway's (16) argument for a public health approach to gun violence that is focused on prevention, broad and inclusive in its exploration of interventions, and shared responsibility over blame. Following the spike in gun violence that coincided with the COVID-19 pandemic, and the crisis in the legitimacy of policing that occurred in 2020, there has been increased interest in CVI as a component of community safety (17).

In 2021, our research team, led by Dr. Shani Buggs, conducted a study to support growing interest and investment in CVI. This research, which resulted in a report entitled *Implementing Outreach-Based Community Violence Intervention Programs: Operational Needs and Policy Recommendations* (2022), was based on interviews with leaders and practitioners in the field.^1^ Our 15 interviewees had an average of 20 years of experience in CVI organizations across eight major U.S. cities. They had expertise in using both well-known CVI models, such as Cure Violence (18), and other promising and evidence-informed strategies that were developed and evolved based on community needs and leadership insight. Our research engaged their insights about challenges, successes, needs, and recommendations in the implementation and operation of outreach-based CVI programs. These outreach-based program models rely on individualized intervention strategies that are co-implemented with a network of community organizations. Through the identification of individuals at the highest risk of violence involvement, these programs entrust mentors with specialized skills and experiences to form relationships, guide personal, and professional development, and ultimately help to mitigate the potential for future violence to occur (19). While models differed across communities, these organizations faced similar challenges, and we derived common themes from their frameworks and approaches.

In our conversations with CVI practitioners, we learned that changing narratives around community violence is a central necessity for the field of CVI to advance and save lives. Specifically, CVI professionals spoke to the necessity to counter entrenched narratives that demonize individuals who have been involved in violence and justify their punishment, isolation, and removal from familial and communal networks. As we listened to these leaders, we heard narratives that reflected empathy and understanding, connection and positive relationships, repair, and redemption as central tenets of outreach-based community violence intervention and its potential to transform and save lives. We learned about alternative forms of conflict resolution and accountability that can interrupt future violence, reflect the needs of those who have been harmed, and humanize all parties who have been involved in or affected by violence. These narratives recognize that the overwhelming majority of perpetrators of harm have themselves been victims (20, 21) and that every human is more than their worst transgression (22). CVI professionals concurred on the urgency of challenging dominant narratives that shape how we understand violence, its causes, and its appropriate responses.

In this perspective article, we identify transformative narratives, values, and practices that are offered directly and indirectly by the field of CVI to prevent and respond to violence. Bridging the fields of human geography, African American studies, sociology, public health, and public policy, we leverage our collective expertise to develop a transdisciplinary perspective on policing and carceral logics, with a shared understanding of their roots in structural racism and violence (23). Through a literature review motivated by the themes that emerged in our interviews, we identify and contextualize narratives that support policing and incarceration, including their basis in logics of retribution, isolation, and counterinsurgency, following entrenched fault lines of white supremacy and racial capitalism. Against these logics of policing and incarceration, we identify counter-narratives that emerged in our analysis of CVI programs. These narratives underline the value of enhancing resource accessibility; combating poverty; fostering positive relationships among individuals and community networks; and building political capacity to prevent, respond to, and heal from violence.

We conclude that formally and systematically adopting these tenets of outreach-based CVI has a strong potential to contribute to undoing the violent and ideologically entrenched logics of policing that perpetuate poverty, systemic abandonment, and trauma while building the conditions for safety. Given this critical moment in time, when our society is grappling with choices around the most appropriate modes of response to elevated rates of interpersonal gun violence and continued maintenance of the status quo through state-sanctioned violence, we see an important opportunity to leverage learnings from within communities that are most affected by violence and to change narratives around responses to violence to influence policies and practices that address it.

## Logics of policing and incarceration

The ideology that policing and incarceration are appropriate and effective at preventing violence is deeply entrenched in the US. This ideology is upheld and reproduced in a wide array of media that uncritically privilege the narratives and perspectives of law enforcement, from journalism to entertainment (24–27). These forms of media affirm the widespread belief that policing and incarceration are invested in—and effective at—promoting safety. This belief is further entrenched as practices of policing, if not the physical presence of law enforcement officers, are incorporated and taken for granted in a wide range of institutions, including K-12 schools (28), hospitals and other healthcare settings (29), public parks and recreational spaces (30), and child welfare and other social services (3).

Scholars across an array of disciplines have worked to counter these beliefs, demonstrating that rather than promote health and safety, the central role of policing is to preserve the interests of capitalism and white supremacy. U.S. institutions of policing and incarceration emerged to exert the violence, discipline, restraint, and confinement needed to maintain settler-colonialism and chattel slavery (31–34). These institutions adapted to facilitate forced labor as punishment for crimes after the abolition of slavery with the passage of the 13th amendment (35, 36). They adapted further as incarceration rose to massive proportions in the 1980s and 1990s, due to phenomena including the War on Drugs and tough-on-crime policies such as mandatory sentencing and “three strikes” laws. This expansion led to a 500% increase in the prison population since 1970 (37, 38) and the per capita incarceration of U.S. residents at a drastically higher rate than any other country in the Global North (31, 39). Despite these astounding investments in policing and incarceration, the US is remarkably unsafe; Grinshteyn and Hemenway (8) demonstrated homicide rates to be seven times higher in the US than in other countries in the Global North.

Informed by this body of scholarship, we argue that institutions of policing and incarceration are not fundamentally designed to reduce violence or promote health and safety. As such, increased investment through the expansion of existing capacities and attempts to reform is ill-fated to address violence as a nationwide public health crisis. In demonstrating this, we argue that the central logics of policing and incarceration—retribution, isolation, and counterinsurgency—each rely on a claim to legitimate, lawful violence that is detrimental to public health. In doing so, we lay the groundwork to consider alternative logics offered by outreach-based CVI and informed by public health to prevent violence including harm reduction, person-centered models for responding to human crises, and systemic and structural transformations. These logics and values point toward a future of violence reduction and prevention that divests from the harmful logics of policing and incarceration.

### Retribution

A central logic of systems of policing and incarceration is that retribution—revenge or punishment—is an appropriate response to harm. Retribution can take the form of force, discipline, incarceration, torture, or death. Rather than responding to the needs or desires of individuals who have been harmed or whose loved ones have been harmed, retribution is enforced in the name of the state without regard for these individuals or families (40). The processes of determining and inflicting punishment often run counter to the wishes of the survivors of violence, as research has shown that most survivors of violent crime favor rehabilitation over punishment (41). Retribution simultaneously fails to meet the needs of co-victims of violence, including families of homicide victims, who are often further traumatized or endangered by involvement in the criminal legal system (42–46).

This rejection of punishment and fear of further harm is reflected in the fact that less than half of those harmed in violent crimes report them to the police (47). As Danielle Sered (48) put it, “more than half the people who survive serious violence prefer *nothing* to everything offered by law enforcement.” By failing to recognize the humanity of the person accused of causing harm (49), the underlying drivers that led to the harm in the first place (48), or the harmed's desire for authentic accountability and restoration of safety (41), retribution fails to address societal structures that contribute to the likelihood of violence or to guide individuals away from resorting to violence in the future, fueling cycles of violence rather than preventing future harm.

### Isolation

A second foundational practice of policing and incarceration is the extended and extreme isolation of individuals from their families, support networks, and neighborhoods through incarceration and detention. Isolation is imagined to promote safety by separating deviant individuals from the rest of society (39). This idea is premised on the existence of an outside society that would be safe, non-violent, and healthy if it were not plagued by criminals, who are often portrayed as “bad people” by nature and thus not worthy of dignity and humane treatment. This dualism—between responsible citizens and dangerous criminals, between good and bad people—has been produced and maintained through racist and xenophobic ideologies in which racial and ethnic others are seen to threaten an idealized white public (50–52). This logic of the isolation of deviant individuals obscures the structural realities through which violence pervades U.S. society. These structural realities include a lack of safety, a prevalence of trauma and an absence of resources for rehabilitation, and poor health outcomes for those both inside and outside of the walls of prisons, jails, and detention centers.

When individuals are incarcerated in prisons, jails, and detention centers, they can no longer participate in earning a living and supporting children or families. They are subjected to degrading, inhumane, and unhealthy conditions and are frequently victimized and re-traumatized in jail or prison, exiting with worsened mental health (48, 53). Upon release, they may return to similar or worse conditions of danger, poverty, deprivation, and housing insecurity (54). They are further faced with minimal opportunities for civic engagement, alongside a lack of access to legal, liveable-wage employment, and education (39, 55). These are all factors that reproduce the conditions underlying community violence.

### Counterinsurgency

A third and central logic of policing and incarceration is counterinsurgency. Counterinsurgency is the neutralization of the political capacity, activism, self-determination, and creation of support networks in communities that are seen to threaten state power and racial capitalism. Efforts to promote health, combat poverty, and build political capacity in Black and brown neighborhoods and communities have been treated as threats to be neutralized by counterinsurgency practices.

Rooted in efforts to contain slave rebellions, indigenous sovereignty, and immigrant solidarity, practices of counterinsurgency evolved and expanded in response to the gains of Black Power and Civil Rights movements of the 1960s and 70s (56, 57). In one particularly illustrative example, during the Vietnam War era, the C.I.A. characterized the Black Panther Party's Free Breakfast program—in which community members pooled resources to provide breakfast to youth in poverty—as “the greatest internal threat to national security” [(58, 59), p. 123]. SWAT teams and other militarized techniques of domestic policing were developed in response to the building of these kinds of communal support systems and political empowerment. By treating these forms of political action as internal threats on par with external threats to state power, practices of counterinsurgency involved the infiltration and surveillance of community groups, raids on community offices and gathering places, assassinations of political leaders, and the taking of political prisoners (59).

Counterinsurgency efforts and their associated forms of militarized policing have continued to neutralize community networks in Black and brown communities, as tactics and machinery developed in the War on Terror have been implemented against community building and political action in the 21st century (60). Counterinsurgency neutralizes political capacity and community support networks, and in doing so, reproduces poverty, scarcity, and trauma.

## Logics of community violence intervention

Outreach-based CVI rejects the logics that bodily and psychological harm, extended and extreme isolation and removal from communities, and communal divestment and disempowerment are appropriate responses to violence. Rather than using punitive models of justice that can reproduce harm and trauma through dehumanizing treatment and disregard for collateral consequences, outreach-based CVI programs seek alternative forms of harm reduction and accountability that increase safety for the individuals involved in violence and their communities and that are responsive to the needs and desires of individuals who have survived or who have lost loved ones to violence. These accountability practices may include taking responsibility and acknowledging the impact of one's actions, apologizing and expressing remorse, working to repair the harm done, and ceasing to commit similar harm (48). Accountability practices are oriented toward the prevention of further harm from being perpetrated and toward the healing of individuals and communities (61, 62).

While accountability processes are important to any response to harms done, CVI programs also work relentlessly to prevent violence from occurring in the first place. This focus on prevention is a critical distinction between the approaches of policing and the criminal legal system, which respond to harms that have already occurred. To prevent violence and to improve the life chances of individuals who have been involved in violence, the central practices of outreach-based CVI programs involve building positive relationships and social safety nets, fighting poverty and increasing access to resources for healthy and fulfilling lives, and building political capacity for individuals and communities caught in cycles of violence to transform their conditions and environments.

### Relationships

Building positive relationships and safety nets at various scales is the fundamental strategy of outreach-based CVI (63). A central part of this strategy is the creation of one-on-one connections that model trust, reliability, and safety between outreach workers and participants, within an environment that responds to challenges in healthy relationship building and maintenance. Recognizing that relationships characterized by trauma, fear, and anger are at the center of violence, CVI professionals work to demonstrate that other forms of relationships are possible and available.

In addition to one-on-one relationships, outreach-based CVI programs also seek to build positive relationships within family units, within communities, and between neighborhoods. The practices and values of relationship building can involve responding directly to violence through vigils and mediated community conversations. They can also include pro-actively promoting community bonds of trust, familiarity, and acknowledgment through events such as sports tournaments and block parties. By centering the importance on personal connections and proximity, rather than extraction or isolation, these values align with research that strengthening positive and supportive relationships can directly or indirectly lead to decreased risk of violence (64). By building a support system for individuals in the context of larger support networks for families and neighborhoods, outreach-based CVI strategists recognize that it takes a “village”—a multi-layered system of supports and safety nets—for any individual to pursue a safe and fulfilling life (65).

### Resources

Resource deprivation, concentrated poverty, and income inequality have correlated with violence across U.S. cities (66–72). Outreach-based CVI professionals recognize that poverty is a major underlying factor in community violence and that connecting individuals, families, and extended support networks to resources is a fundamental building block for the cessation of violence. They agree that access to the resources for a healthy life is imperative for violence intervention efforts to succeed, challenging retributive approaches to resource allocation that reproduce policing by assessing individuals for their deservedness of resources. Much of the work that outreach-based CVI professionals described in our research involved helping their participants gain access to needs easily and unconditionally, including housing, employment, and legal documents; competent, non-punitive systems for physical and mental health, childcare, education, and conflict mediation; and navigation of social services and legal systems (63). They emphasized the potential of strategies to alleviate poverty and promote access to resources in their cities and communities to stem the violence.

### Political empowerment

Ensnarement in the criminal legal system reduces the ability of individuals and their families to connect, build positive networks, and engage in political action with their communities. Households are taxed financially and emotionally as they must extend from their neighborhoods to courtrooms and visiting rooms, while intensive policing and state presence degrade the informal networks that promote social wellbeing and stability (39, 73). Individuals who are incarcerated are limited in their ability to build networks and shape their environments and their lives, both when they are behind bars and as they work to rebuild their lives in the aftermath.

Political capacity is built through strong social networks and investments of time, energy, and resources. The start-up and operation of outreach-based CVI organizations can contribute to the building of political capacity by harnessing the informal networks of information, influence, and care to respond to violence and interrupt further harm. In doing so, they contribute to building the relationships, trust, and communication that the persistence of community violence and intimidation of policing and incarceration can degrade. Valuing their communities' self-determination and autonomy, they aim to break down information silos, create channels of communication, and build relationships among organizations to increase their ability to empower individuals and positively impact lives. This ethic of empowerment is sustained by those who are directly impacted and therefore most invested in community care and the cessation of violence.

## Conclusion

A dramatic change in entrenched narratives and logics about public health and safety is necessary for meaningful reductions in violence. Such a change requires a rejection of the logics of policing and incarceration that rely on subtractive practices that primarily seek to punish, dehumanize, and isolate individuals from communities and networks of support, and that undermine community building and political power. These logics are underpinned by the myth that the widespread persistence of violence in the U.S. is the fault of inherently deviant individuals and aberrations in an otherwise safe, civil, and functioning society. Such logics miss the violence that pervades all facets of a society structured by racial capitalism and white supremacy. They also run counter to both the scholarly work across various disciplines and the experiential wisdom of communities most impacted by the violence that suggest alternative logics are necessary for consequential and persistent reductions in community violence.

The practices and philosophies of outreach-based CVI programs offer powerful rebuttals to these logics of policing and incarceration. CVI practices reject the subtractive logics of punishment, extreme isolation, and disenfranchisement, instead pursuing an additive definition of safety based on creating positive relationships, fighting poverty and increasing access to resources, and building political capacity alongside mutual aid networks and safety nets. Against the entrenched logics that dehumanizing practices of isolation and retribution through policing and incarceration can prevent violence, outreach-based CVI has been shown to reduce harm by strengthening relationships to keep communities whole, celebrating community building as a promise rather than a threat. As a society, we have the potential to elevate the language, narratives, and values of outreach-based CVI to transform our responses to violence and foster safer communities.

## Data availability statement

The original contributions presented in the study are included in the article/supplementary material, further inquiries can be directed to the corresponding author.

## Author contributions

All authors listed have made a substantial, direct, and intellectual contribution to the work and approved it for publication.

## Footnotes

^1^These interviews were semi-structured and iterative, building on knowledge and expertise shared in the early stages to adapt as saturation was achieved on various topics. The interviews were conducted virtually following verbal consent, and the project was deemed “exempt” by the University of California, Davis, Institutional Review Board Administration.
